# “It was complete chaos day and night and no one was able to help”: A qualitative study on the support needs of family members exposed to violence by patients with brain tumors

**DOI:** 10.1093/noajnl/vdag077

**Published:** 2026-04-03

**Authors:** Amina Guenna Holmgren, Annika Malmström, Eskil Degsell, Johanna Simmons, Lisa Kastbom

**Affiliations:** Department of Neurobiology, Care Sciences and Society, Karolinska Institutet, Stockholm, Sweden; Clinical Department of Geriatrics and Palliative Medicine in Linköping, Region Östergötland, Linköping, Sweden; Division of Cell and Neurobiology, Department of Biomedical and Clinical Sciences, Linköping University, Linköping, Sweden; Swedish Brain Tumor Association and NOCRiiC, Neuro Oncology Clinical Research, Innovation, Implementation and Collaboration, Karolinska University Hospital, Stockholm, Sweden; Department of Micro, Tumor and Cell Biology (MTC), Karolinska Institutet, Stockholm, Sweden (E.D.); Clinical Department of Geriatrics and Palliative Medicine in Linköping, Region Östergötland, Linköping, Sweden; Department of Health, Medicine and Caring Sciences, Linköping University, Linköping, Sweden; Department of Health, Medicine and Caring Sciences, Linköping University, Linköping, Sweden; Primary Health Care Center Ekholmen, Region Östergötland, Linköping, Sweden

**Keywords:** behavioral and personality change, child maltreatment, intimate partner violence, primary brain tumor, support needs

## Abstract

**Background:**

This study is part of the Swedish national research project BRAVE (***B****rain Tumor* ***R****elated* ***A****ggression and* ***V****iolence* ***E****xposure*), which has highlighted extensive suffering among family members exposed to violence by brain tumor patients with behavioral and personality changes (BPC). The aim was to explore the needs, support received, and perceived gaps in support among family members exposed to violence by individuals suffering from brain tumor-related BPC.

**Methods:**

Individual interviews were conducted with 25 family members who had experienced violence from a brain tumor patient. The data were analyzed using qualitative content analysis.

**Results:**

Participants stressed the need for a holistic care approach, including private consultations with family members to enable the disclosure of violence by brain tumor patients. An integrated care approach and continuity of care were seen as essential for building trust, identifying violence, and supporting both patients and families. Participants expressed frustration with the healthcare system’s rigid focus on patient autonomy, even in cases where patients lacked cognitive capacity, leaving families without adequate information and support. They expressed that family members exposed to violence by brain tumor patients had complex needs for support.

**Conclusions:**

The support needs of family members exposed to violence by individuals with brain tumors are multifaceted, and interventions must be tailored to specific circumstances. Addressing these needs requires coordinated efforts from multiple stakeholders. An intervention that provides appropriate support for patients, family members, and staff who encounter them is urgently needed.

Key Points

- Improved detection of brain tumor-related violence is needed.
- One-on-one interactions enhance disclosure of brain tumor-related violence.
- A holistic approach to brain tumor care must include family members.

Importance of Study

Violence perpetrated by individuals with brain tumors remains an important and unexplored area of research. Insights from the Swedish Brain Tumor Association, clinical experience, and findings from our previous research suggest that such violence can cause significant suffering among family members. These findings underscore the urgent need for a deeper understanding and tailored interventions to help families facing the dual burden of caregiving and exposure to violence. Currently, there are no established guidelines for providing support to this group. Furthermore, due to the complex nature of the situation, the needs of the group often fall outside the scope of the healthcare system, social services, and legal system. The overarching aim of this study is therefore to serve as part of a sound bases for developing an intervention that provides appropriate support for brain tumor patients, their families affected by violence, and the professionals who encounter them.

Patients diagnosed with brain tumors may exhibit behavioral and personality changes (BPC), such as impaired impulse control or aggressive behavior. These changes can emerge as early indicators of the disease and may persist or evolve throughout its progression, significantly affecting both the patients and their family members.^1-3^ Although research in this area remains limited, existing studies suggest that BPC are often challenging to identify and treat effectively.^1-3^ Certain clinical situations in brain tumor patients can contribute to an increased risk of BPC, such as frontal or temporal involvement,^4-6^ mass effect or edema,^7^ treatment with corticosteroids,^1^^,^^8^ use of antiepileptic drugs,^1^^,^^8-10^ perioperative factors,^11^ and disease progression.^1^

Previous studies have shown that family members of brain tumor patients are at high risk of caregiver burden and distress.^12-15^ The disease is associated with a substantial symptom burden, frequently involving cognitive decline. As a result, patients gradually lose their independence and become increasingly dependent on family members for everyday support.^16^ This dependency can lead to shifts in roles and relationships, and may impose significant emotional, psychological, and financial strain on family members as caregivers.^17-22^ Extensive research has highlighted the significant support needs of individuals diagnosed with brain tumors and their families.^23-25^ Due to changes in life circumstances associated with the illness, family members frequently experience unmet needs for information, as well as practical and emotional support.^26^^,^^27^ In cases of rapid disease progression, family members may have little time to adapt and develop effective coping strategies.^22^^,^^28^ Additionally, a lack of understanding of the disease and its expected progression can lead to uncertainty among family members about the kind of support they need. Previous research has emphasized the need for improved information and education to help family members adapt to changing circumstances and roles.^23^ Additionally, it has been suggested that family members should have more frequent contact with healthcare professionals, as support needs may shift rapidly as the disease progresses.^18^

In cases where family members are subjected to violence by brain tumor patients with BPC, the situation becomes particularly complex. Research specifically addressing violence perpetrated by individuals with brain tumors is limited. However, experiences from the Swedish Brain Tumor Association, clinical observations, and our previous study^29^ indicate that such violence can cause significant distress among family members. These findings underscore the urgent need for a deeper understanding and tailored interventions to help families facing the dual burden of caregiving and exposure to violence. The World Health Organization (WHO) defines intimate partner violence (IPV) as “behavior within an intimate relationship that causes physical, sexual or psychological harm, including acts of physical aggression, sexual coercion, psychological abuse, and controlling behaviors.”^30^ This definition includes violence perpetrated by both current and former spouses or partners. Child maltreatment is the abuse and neglect that occurs in children under 18 years of age in the context of a relationship of responsibility, trust, or power.^31^

Our previous study^29^ revealed that most participants expressed that they were convinced that the affected individual would not have displayed such aggressive or harmful behavior if it had not been for the BPC related to the tumor. A few participants, however, suggested that certain tendencies were present beforehand and seemed to be intensified by the tumor. The family members exposed to violence from individuals with brain tumors often choose to remain in their caregiving roles, reporting a strong sense of obligation to provide care despite the violence. These situations often give rise to ethical dilemmas for both families and healthcare professionals, where the obligation to respect patient autonomy conflicts with concerns about the patient’s capacity to make informed and sound decisions.

The present study is the second part of the research project BRAVE (***B****rain Tumor* ***R****elated* ***A****ggression and*  ***V****iolence*  ***E****xposure*), investigating violence in relation to brain tumors from different perspectives. The aim is to explore the needs, support received, and perceived gaps in support among family members exposed to violence by individuals suffering from brain tumor-related BPC, primarily in patients with glioma. The perspectives of both intimate partners and children are included. As this is a qualitative study, the focus is to gain an in-depth understanding, and the findings are intended to provide an exploratory foundation for future research.

## Methods

### Design

A qualitative descriptive approach^32^ was used to explore the perceived needs and gaps in support among family members exposed to violence by persons suffering from brain tumor-related BPC. To ensure methodological rigor, the study followed the Consolidated Criteria for Reporting Qualitative Research (COREQ) guidelines.^33^

### Setting and Participants

Participants were recruited through purposive sampling^34^ by advertising the study via the Swedish Brain Tumor Association and several clinics caring for brain tumor patients. Inclusion criteria were: family member who had experienced violence from an individual diagnosed with glioma, at least 18 years old at the time of the interview, ability to speak and understand Swedish, and willingness to participate in a recorded interview. Individuals with cognitive impairment or severe psychiatric conditions that could hinder their ability to participate were excluded. Four participants initially provided informed consent, stating that their relative had glioma. This was later updated to a diagnosis of meningioma. Their accounts were also included in the study, as their experiences were considered to align closely with those of the other participants.

### Data Collection

For participant characteristics and interview information, see Table 1. An interview guide (see Appendix S1), developed by the researchers, was used to facilitate conversations. After an initial overview of the study, participants were asked to reflect on their experiences of violence from an individual with a brain tumor and to discuss their needs, the support they received, and the support they felt was lacking. The interviews were semi-structured and included open-ended questions. All interviews were conducted by the same researcher (LK: PhD, physician), who had no prior relationship with the participating family members. Participants were asked to share their experiences of support, or lack thereof, mainly from healthcare, but also from society, related to violence. All interviews were digitally recorded and transcribed verbatim by an external transcription service.

**Table 1.** Descriptive characteristics of the 25 participants and details of the interviews

	*n*	%
Age at the time of interview		
Mean (years; range)	50 (21-75)	
Median (years)	56	
Gender		
Female	20	80
Male	5	20
Relation to the brain tumor patient		
Partner	21	84
Child	4	16
Numbers of brain tumor patients to whom the participants were related	21	
Status of the brain tumor patient		
Alive	7	33
Deceased	14	67
Type of brain tumor		
Glioma	20	95
Meningioma	1	5
Duration of illness*		
< 1 year	9	36
1-5 years	11	44
6-10 years	3	12
> 10 years	2	8
Living situation at the time of the interview		
Living with another adult	4	16
Living with another adult and child/children	3	12
Living with child/children	6	24
Living alone	12	48
Participant recruited from		
Healthcare	14	56
The Swedish Brain Tumor Association	11	44
Interview details		
Time of interviews	Feb 2024-Jan 2025	
Duration of interview	58-148 minutes	
Interview setting		
Telephone	9	
Online	9	
Physical meeting	7	

* If patient is deceased: Time from diagnosis until death.

If patient is alive: Time since diagnosis at the time of the interview.

### Analysis

**Figure 1.**
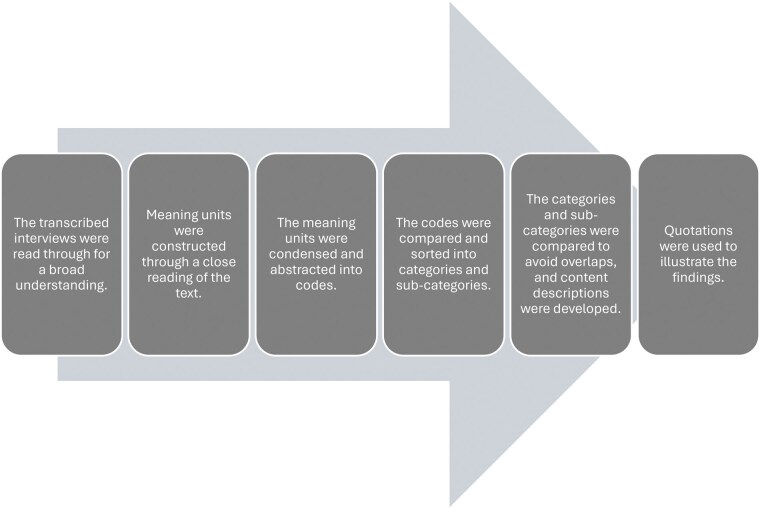
The six steps used in the qualitative content analysis as outlined by Graneheim & Lundman.

The data were previously analyzed with a focus on family members’ experiences of different aspects of violence.^29^ This study explores the data from another perspective, focusing on experiences of support and support needs related to violence. Data were analyzed using qualitative content analysis with an inductive approach, allowing categories to emerge from the material (see Figure 1).^35^ Initially, two researchers with expertise in qualitative methodology (AGH: PhD, registered nurse and LK) independently read all transcripts to gain a comprehensive understanding of the material. Words and sentences reflecting key concepts were highlighted and subsequently labeled with codes. These codes were then compared to identify similarities and differences, allowing patterns to emerge. These patterns were organized into clusters of subcategories, which were further refined into broader categories. The development of categories involved a continuous, iterative process, moving back and forth between the full text, codes, subcategories, and categories.

## Results

**Figure 2.**
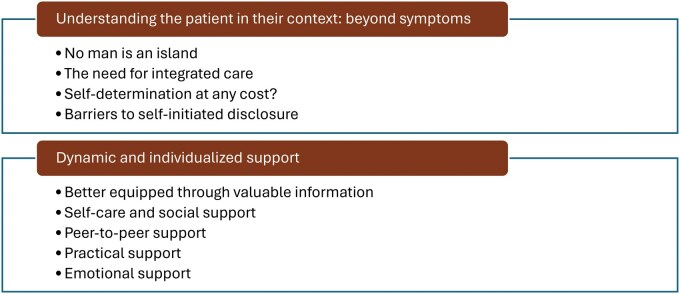
Overview of the two categories with nine sub-categories capturing the support needs and perceived support of family members exposed to violence by individuals with brain tumor related behavioral and personality changes (BPC).

When analyzing the data, two categories emerged that describe family members’ needs, the support they received, and the perceived gaps in support related to violence associated with brain tumors: *Understanding the patient in their context: beyond symptoms* and *Dynamic and individualized support.* In total, these categories contained nine sub-categories (Figure 2). Table 2 summerizes the categories and sub-categories with example quotations.

**Table 2.** Summary of the categories and sub-categories with example quotations

Quotation	Sub-category	Sub-category
“So the whole family is very affected by it [the brain tumor]. And everyone also understands what can happen to the patient, that they can change and such, that it is probably due to that.”	No man is an island	Understanding the patient in their context: beyond symptoms
“I needed someone the whole time. The one who was there from day one. I needed her to hold my hand from the beginning. Not that the ties were cut when X [name of partner with brain tumor] was moved to another department and she was no longer allowed to take care of me or talk to me. I really needed… When I was the most alone and completely… The world was completely upside down and no one could help.”	The need for integrated care	
“If you are not capable of understanding the illness you suffer from, then I don’t think you can make rational decisions either… Then I think it would be reasonable to have a kind of … an option to transfer responsibility to someone who can have a better, more informed view of the matter.”	Self-determination at any cost?	
“I froze when he came near me. And then he stood behind me and slammed his fist down on my left shoulder. And I thought, ‘oh, I … I’m not going to scream’… Then the nurse said, ‘I see,’ and again, “I see.” And then the tears came, because someone else acknowledged [me] and told me, “I see how you feel.”	Barriers to self-initiated disclosure	
“But you feel very alone, and all this shame and guilt and a bad conscience. And I’m not the one who is sick, so… That means that you can have a hard time opening up. Then it may have been easier if you had found out that ‘this, this could happen. It might be this way…’ Then you’re also prepared for that, and then it might be easier to open up when it does happen, and seek help.”	Better equipped through valuable information	Dynamic and individualizedsupport
“Then I found the courage… I went and told our neighbors how bad things were. Then they came over several times a day. They all came… It was about the mail and everything else [laughter]. It was about anything you can imagine, to check how things were going.”	Self-care and social support	
“And that’s when I first realized that I’m not alone in this. There are more people who are going through the same thing, that is, this aggression that manifests itself in different ways. But it was the first time that it really hit that this is happening to others too. It’s not just XX [name of partner with brain tumor] who has become really strange, it is because of the disease. That was probably the first time it dawned on me.”	Peer-to-peer support	
“ Well, then at the oncologist they said that… well, those who… Or, if things become completely unmanageable, then you can… so dad could move somewhere else and live there, but I really didn’t want that.”	Practical support	
“I think that’s the saving grace actually, having someone to talk to who can listen and who I can tell almost anything. She doesn’t judge… She just listens to me and adds valuable insights. So that’s… That’s where I get to let it all out.”	Emotional support	

### Understanding the Patient in Their Context: Beyond Symptoms

#### No man is an island

­—Participants expressed that healthcare providers primarily focused on the patient, while the family as a unit was often overlooked. Many described a lack of recognition and exclusion from the care process. They felt that they were not given space to ask questions or express their concerns. They emphasized the importance of being recognized and acknowledged as a family member, along with the need for private, individualized conversations with healthcare staff. This need became particularly relevant in situations involving BPC or violence. In these circumstances, family members often felt isolated and unsupported, facing emotionally and physically challenging conditions at home.

Interviewees suggested holding joint medical appointments with physicians in the presence of a dedicated oncology nurse at the clinic, as well as offering the option of separate consultations with the patient and family members before or after these visits. Individual meetings with healthcare staff were also seen as important, as they provide a safer space for family members to disclose experiences of violence.

Interviewees expressed a strong desire for a more holistic approach where the entire family is considered. This included identifying and addressing the needs of children, for example, by involving child trauma teams.

So the whole family is very affected by it [the brain tumor]. And everyone also understands what can happen to the patient, that they can change and such, that it is probably due to that.

(Participant LK9. Partner)

Participants suggested that appointing a dedicated oncology nurse, not only for the patient but also for family members, with knowledge of both the disease and the patient’s individual circumstances, could facilitate communication about experiences of violence and improve the coordination of support. It was also suggested that home visits could be valuable for gaining a more accurate understanding of the family’s unique situation and needs. This could also offer professionals a chance to observe potential signs of violence that might not be visible in a clinical setting. While the patient′s physical symptoms received more attention, participants noted that cognitive and psychosocial issues were frequently left unaddressed. Those who had access to counselors at the hospital felt these professionals better understood the family’s situation compared to counselors in schools or youth clinics. Overall, participants expressed that, in addition to care from physicians, they would benefit from access to professionals who could recognize and address the broader psychosocial impact of the illness on the entire family.

#### The need for integrated care

—Participants emphasized the importance of a consistent care relationship throughout the illness trajectory. Many described a lack of continuity, particularly in connection with department transfers, when oncology nurses, counselors, and physicians were frequently replaced. This led to frustration due to a perceived lack of coordination and responsibility. When BPC or violence occurred, the absence of continuity was seen as especially problematic, as it made it more difficult for family members to seek help, build trust with healthcare staff, and raise concerns in a safe and supportive environment.

I needed someone the whole time. The one who was there from day one. I needed her to hold my hand from the beginning. Not that the ties were cut when X [name of partner with brain tumor] was moved to another department and she was no longer allowed to take care of me or talk to me. I really needed… When I was the most alone and completely… The world was completely upside down and no one could help.

(Participant LK7. Partner)

Participants underlined the importance of being acknowledged and understood by a healthcare professional with a sustained relationship with the family. This reduces the emotional burden of repeating their story and makes it easier to engage in difficult conversations about violence. Continuity created trust and emotional security, especially when the same physician, nurse, or counselor remained involved during the entire disease trajectory, sometimes even beyond the death of the patient. Conversely, frequent changes in staff—especially counselors or psychologists—created confusion, disrupted trust, and left participants feeling unsupported.

A recurring theme was the absence of a single professional with overall responsibility, rather than focusing on isolated tasks based on specific medical competencies. Family members expressed a desire for someone to accompany them throughout the entirety of the care journey. Those who experienced continuity in their interactions with healthcare professionals described this as a source of stability and reassurance. However, this was not the norm.

You have to see it as a journey, from the patient’s… and what is the best way to provide information and create continuity in that journey. And these different scenarios, what can happen and what happens when you… I think that. It’s probably where… where you should… Regardless of whether there is violence.

(Participant LK12. Partner)

Sometimes, interventions such as child trauma teams were perceived as artificial or ineffective, particularly when they lacked insight into the specific challenges of brain tumor illness, including associated BPC and violence. In contrast, counselors within oncology settings were valued for their holistic perspective and understanding of the family’s situation. Overall, participants expressed a desire for a care model characterized by continuity, trust, and coordination that would facilitate opportunities for family members to disclose experiences of violence.

#### Self-determination at any cost?

—A tension emerged between maintaining patient autonomy and providing adequate support to family members. Participants voiced frustration with the rigid focus on patient autonomy, even in situations where family members felt that the individual lacked the capacity to make informed decisions.

If you are not capable of understanding the illness you suffer from, then I don’t think you can make rational decisions either… Then I think it would be reasonable to have a kind of … an option to transfer responsibility to someone who can have a better, more informed view of the matter.

(Participant LK7. Partner)

Participants described ethically and emotionally complex situations in which they felt compelled to act against the patient’s wishes in order to ensure safety, both for the patient and for themselves. Situations were described where healthcare providers advised family members to avoid staying at home due to potential danger, while simultaneously informing them that the criteria for involuntary commitment had not been met.

Patients’ refusal to involve family members left them without guidance or support from healthcare, social care, and civil society. When patients declined to receive information, family members were also denied access. While some healthcare providers reinforced this exclusion, others attempted to acknowledge the family members’ perspectives and advocate for their right to be included.

#### Barriers to self-initiated disclosure

—Participants generally expressed a wish to be asked about their circumstances, particularly concerning experiences of violence. However, some expressed ambivalence, uncertain whether they would have shared their experiences if given the opportunity. This was the case even when participants acknowledged the importance of early intervention, as living in fear was emotionally draining. Even when participants were willing to share information regarding violence, initiating the conversation was described as challenging.

Several participants recalled situations in which healthcare providers appeared to be aware of their emotional distress but failed to ask questions. As a result, many felt that no one had a general interest in their well-being. In contrast, some caregivers responded to non-verbal cues by asking direct questions about violence, which participants perceived as both validating and supportive. Several participants emphasized the importance of being asked appropriate questions at the right time, as this increased their willingness to disclose experiences of violence. They stated that if such questions had been asked with an awareness of and sensitivity to their situation, they would have responded openly and honestly.

Follow-up communication, such as regular phone calls from healthcare providers, was perceived as valuable, though participants expressed a desire for more proactive engagement and offers of support. On certain occasions, physicians inquired about potential violence, and counselors investigated the origins of unexplained bruises. Despite a willingness to listen, participants noted that some healthcare providers did not act on the information they shared, leaving them feeling ignored or dismissed. In some cases, participants expressed that no specific support interventions were needed when experiences of violence were disclosed. Rather, the opportunity to speak with someone who listened attentively and asked exploratory questions provided the support they needed. On other occasions, participants perceived that healthcare professionals avoided the topic of violence.

In some cases, healthcare professionals were seen as playing a crucial role in recognizing and addressing violence, with a particular sensitivity to indicators of emotional distress.

I froze when he came near me. And then he stood behind me and slammed his fist down on my left shoulder. And I thought, ‘oh, I … I’m not going to scream’… Then the nurse said, ‘I see,’ and again, ‘I see.’ And then the tears came, because someone else acknowledged [me] and told me, ‘I see how you feel.'

(Participant LK18. Partner)

The ability of healthcare professionals to pose direct questions was identified as a critical facilitator in enabling the disclosure of violence. Homecare providers, ambulance staff, and counselors played a crucial role in identifying indicators of violence and collaborating with family members to ensure safety. Participants also emphasized the important role of other actors, such as school staff, in identifying and responding to signs of violence.

### Dynamic and Individualized Support

#### Better equipped through valuable information

—Participants reported receiving detailed information about the tumor’s location, associated symptoms, and potential deterioration, but noted a lack of information about BPC and its potential consequences.

But you feel very alone, and all this shame and guilt and a bad conscience. And I’m not the one who is sick, so… That means that you can have a hard time opening up. Then it may have been easier if you had found out that ‘this, this could happen. It might be this way…’ Then you’re also prepared for that, and then it might be easier to open up when it does happen, and seek help.

(Participant LK2. Partner)

Many felt overwhelmed by the written information provided, especially when it was not followed by a discussion. In addition, participants emphasized that no amount of preparation could replace the value of their lived experience. The complexity and variety of symptoms often made it challenging for healthcare professionals to offer specific guidance, resulting in information that was frequently too general. As a result, participants often independently sought supplementary information, particularly through online sources.

Participants requested practical information about BPC and where they could seek help and support. They sought clear explanations regarding risks, safety precautions, and prognosis, including how long their current situation was likely to continue. Although some were aware of the potential for BPC, they often lacked detailed knowledge. While healthcare providers sometimes described possible scenarios, participants expressed a wish for more concrete guidance. They expressed a wish for a clear definition of violence and its implications, and highlighted the need for proactive information about the relationship between brain tumors and BPC, including violent behavior. Receiving such information at an early phase was seen as essential. Understanding that BPC could result from medication as well as the tumor itself was also considered important.

Participants reported receiving inconsistent guidance regarding the management of potential violence. Nevertheless, family members indicated that receiving clear and specific advice contributed to a sense of reassurance. Many felt that it should be the responsibility of healthcare professionals to proactively raise the issue of violence as a potential risk, rather than expecting patients or family members to initiate such conversations.

If you start talking about it from the healthcare side, then it’s not so taboo. It is much worse to have to take the initiative yourself as a relative and say ‘hey, here I am, I am afraid for my husband, I am afraid for my wife, I am afraid for my son, my daughter, my father.’ It is awful.

(Participant LK1. Partner)

Participants also highlighted the value of forums for family members and opportunities to have conversations with physicians about possible symptoms.

#### Self-care and social support

—Self-care practices were seen as essential, particularly in managing the emotional and psychological impact of living with or being exposed to violence. Physical activity played a particularly central role, especially when the home was no longer seen as a safe space. Creative and social environments, such as choir groups, sports, and time with friends, were described as helping participants maintain a sense of normality and control. Work became a sanctuary where they could feel competent and reconnect with normal, everyday routines. The structure and social contact at work helped them temporarily escape from stress. Participants described varying degrees of support from friends and family members. Practical support, discreet support from neighbors, and regular check-ins from managers and colleagues were particularly appreciated.

Then I found the courage… I went and told our neighbors how bad things were. Then they came over several times a day. They all came… It was about the mail and everything else [laughter]. It was about anything you can imagine, to check how things were going.

(Participant LK18. Partner)

While participants often received support from those around them, many expressed disappointments regarding the lack of support from healthcare. Instead, friends and family often served as the primary source of help and support.

#### Peer-to-peer support

—Participants described social media groups as a significant source of support, offering a sense of solidarity with others facing similar circumstances.

And that’s when I first realized that I’m not alone in this. There are more people who are going through the same thing, that is, this aggression that manifests itself in different ways. But it was the first time that it really hit that this is happening to others too. It’s not just XX [name of partner with brain tumor] who has become really strange, it is because of the disease. That was probably the first time it dawned on me.

(Participant LK1. Partner)

Many participants highlighted the importance of connecting with others in similar situations, whether online or in person. Sharing experiences allowed them to make sense of their situation. Through these interactions, participants reported learning about BPC and its connection to the illness, and many found comfort in being able to share their feelings openly. The opportunity to talk openly with someone who understood their experience was perceived as healing, particularly since discussing such issues outside these groups was often associated with feelings of shame. While these forums offered participants a way to process experiences, some found it emotionally difficult to engage, particularly when reading about the traumatic experiences of others. For others, the emotional intensity of these accounts was overwhelming, ultimately leading them to withdraw from the groups to protect their own well-being. Also, some individuals chose not to participate in these groups because they did not want to focus exclusively on the brain tumor.

I don’t want more cancer in my life, I want less. And I only care about XX [name of partner with brain tumor]. It’s very tragic for everyone who is affected. It’s not that I lack empathy, but right now I want to focus my strength and energy on helping XX. And so, I don’t want to talk about other people’s experiences with cancer.

(Participant LK17. Partner)

#### Practical support

—There was a need for concrete support when family members were no longer able to manage their caregiving responsibilities on their own. Participants reported that healthcare services neither provided sufficient practical assistance nor offered guidance on how to manage daily life, and that healthcare professionals often appeared uncertain about what support was available.

And you wish you had that at your disposal, that you had some kind of training in the expected behavior as a relative. If this happens, don’t do this, but then… Keep your distance. But it’s clear, everyone reacts differently and not all patients become violent and engage in self-harm so… But I wish that were the case, because then you would never have tried to physically stop her from harming herself.

(Participant LK17. Partner)

Participants who used the municipal service designed to support informal caregivers found it very helpful and suggested that more people should be informed about the program. Many learned about the service from other informal caregivers rather than from healthcare professionals. They attributed this to a lack of practical guidance and perceived that healthcare providers did not view these issues as their responsibility. Among the practical challenges were decisions regarding alternative living arrangements or care, particularly in situations where violence was a factor. While deciding to move a partner or parent to a care facility was a difficult decision, it was often followed by feelings of relief and gratitude.

Well, then at the oncologist they said that… well, those who… Or, if things become completely unmanageable, then you can… so dad could move somewhere else and live there, but I really didn’t want that.

(Participant 15. Child)

Many also highlighted how valuable it was when healthcare professionals initiated the conversation about relocation, as it relieved them of the burden of making the decision alone.

#### Emotional support

—Participants emphasized the importance of having access to supportive conversations, which were perceived as essential to their well-being, particularly when coping with the emotional impact of violence or threat of violence. This could be provided by various actors in society (e.g. deacons) not only healthcare providers.

I think that’s the saving grace actually, having someone to talk to who can listen and who I can tell almost anything. She doesn’t judge… She just listens to me and adds valuable insights. So that’s… That’s where I get to let it all out.

(Participant LK11. Partner)

The timing of supportive conversations was critical. Several participants noted that they were not ready to engage in such discussions at certain points but later felt that earlier intervention might have been beneficial. Many stated that they would have benefitted from counseling earlier in the process, such as during appointments with the physician. Some prioritized the care of their loved ones, refraining from seeking support for themselves. Help was frequently sought only after the patient had passed away.

Children often had their own counseling contacts. While views on their effect varied, many saw them as essential, especially when support was provided through home visits. Conversations with counselors and psychologists were experienced as helpful, and ongoing support after a loved one’s death was considered particularly meaningful.

## Discussion

Patients with brain tumors, particularly glioma, face a progressive and fatal illness, frequently accompanied by cognitive impairment from the time of diagnosis that worsens over the course of the disease.^36^ In some cases, healthcare professionals may anticipate a risk of BPC, which could potentially include aggression or violence, depending on tumor location or clinical presentation.^5^ However, the risk of violence is often difficult to anticipate. Our findings suggest that families are frequently not provided with adequate information regarding potential risks of violence—information they perceive as essential for preparing for and managing potentially dangerous situations. This leaves family members unprepared and vulnerable at home.

Our findings also indicate that patients may choose to exclude family members from care decisions, further limiting access to important information about the expected disease course and limiting their ability to seek help for experiences of violence. Healthcare professionals often prioritized respect for patient autonomy, which meant that family members were not always allowed to access information or only received information when the patient was present. Though this approach may be well-intentioned, it can prevent healthcare providers from receiving or providing valuable information that could benefit both the patient and family members. Previous studies have shown that brain tumor patients often have impaired decision-making capacity, often as early as the time of diagnosis.^36^^,^^37^ Moreover, healthcare professionals may overestimate the ability of patients to make informed decisions, which raises ethical and practical questions about when, how, and to what extent assessments of decision-making capacity should influence both care planning and information sharing.^36^

In cases where IPV or child abuse is suspected or disclosed, healthcare professionals face a dual challenge of protecting the patient’s integrity while also ensuring the safety and well-being of those closest to them. There is an urgent need for healthcare systems to identify ethical and practical approaches to involve and inform family members, especially in the case of patients suffering from BPC. This can often be expected to be in the best interest of both the patient and family members who are responsible for day-to-day care. While patient confidentiality must always be respected, many healthcare systems also recognize the importance of supporting family members in their caregiving role. Even if a patient does not wish to involve their family directly, healthcare professionals may still engage with family members in ways that do not compromise patient privacy, for instance, by inquiring about and listening to their concerns, offering emotional support, and providing general information about available resources. This approach respects patient autonomy while acknowledging the broader relational and caregiving context.

Our findings show that family members often felt that healthcare professionals failed to meet their expectations regarding support, despite formal responsibilities for caregiver support outlined in Swedish policy documents.^38-41^ This gap is particularly concerning given that countries like Sweden have well-established policies and legal frameworks for the recognition, involvement, and support of family members of individuals with long-term illnesses. Although the scope and implementation of such support may vary internationally, our results highlight a troubling disconnect between policy and practice, especially in cases involving BPC or violence.

To provide appropriate support, it is first necessary to raise awareness of and acknowledge violence in patients with brain tumors. Disclosing experiences of violence requires trust. Many participants emphasized that healthcare professionals should take the initiative and raise the issue of violence. This is consistent with previous research on IPV, indicating that patients generally appreciate being asked by healthcare providers about experiences of violence.^42-44^ To enable these discussions, healthcare must move beyond fragmented, single-specialty models and develop systems that allow professionals to address the broader context in which patients live, including their families. This also includes assessing tumor-related BPC and responding with appropriate interventions, such as adjusting patient medications, providing psychosocial support for both patients and families, and implementing protective measures for family members. To do so effectively, healthcare professionals not only need awareness of IPV, but also education on referral pathways and available resources.

Several of the findings in this study are in line with previous research on how healthcare systems should respond to women subjected to IPV. As in this study, the need for emotional support and continuity have repeatedly been emphasized.^45^ Studies of female victims of IPV have also highlighted the important role of healthcare professionals in making relevant referrals, guiding patients through complex societal systems, and providing practical support.^45^ However, most participants in this study not only needed the healthcare system to offer referrals, but also to provide direct information and guidance, particularly in relation to the tumor and related symptoms.

Support needs in the case of violence are multidimensional and extend beyond the healthcare system. While healthcare services play a crucial role, our results highlight the importance of broader support networks. Participants identified faith-based organizations, social networks, and peer support groups as important sources of support. Family members often sought these forms of support independently, as they perceived that formal healthcare services were insufficient in addressing their needs. Several participants described the significance of meeting others in similar situations. These individuals were not only a source of emotional support, they offered practical advice on managing everyday life while living with a partner or parent affected by a serious illness. This aligns with previous studies showing that peer support can help individuals prepare for future challenges and cope with their circumstances more effectively.^46^^,^^47^ However, previous research has identified certain challenges associated with peer support.^46^ Encounters with others in similar circumstances can sometimes result in emotional distress, particularly when witnessing the deterioration or death of fellow patients or the effect on their family members. These experiences often prompt reflection on the individual’s own circumstances.^46^ Despite this, peer support has often been shown to be a valuable resource, as it can provide insight grounded in lived experience and help reduce feelings of isolation.^46^^,^^47^ Our findings show that addressing the full range of needs of patients and families affected by brain tumor-related violence requires a coordinated societal response, involving multiple sectors and community-based actors, including healthcare, social services, legal systems, civil society, and patient-led organizations.

### Strengths and Limitations

The diverse characteristics of the participants, including variations in gender, age, living situations, relationship with the brain tumor patient, educational background, recruitment method, and time since diagnosis, enhance the transferability of the findings.^34^ Although the aim of this study was to explore violence perpetrated by individuals diagnosed with glioma, several participants were family members of a patient diagnosed with meningioma. Nevertheless, no notable differences were identified in their reported views compared to family members of glioma patients. The involvement of researchers with qualitative research expertise from diverse professional and disciplinary backgrounds (nurses and physicians specializing in neuro-oncology, neurosurgery, geriatrics, palliative medicine, and primary care), along with experience in the Swedish Brain Tumor Association, contributed to the validation and credibility of the findings.^34^ The researcher who conducted the interviews is a clinically active physician, which may have influenced the participants’ responses. However, the interviewer maintained a neutral stance throughout the research process and was not involved in the clinical care of the participants. Consolidated criteria for reporting qualitative research (COREQ)^33^ were used for reporting the results, ensuring the trustworthiness of our study.^34^

## Conclusion

The support needs of family members affected by violence from individuals with brain tumors are multifaceted and require a nuanced, context-sensitive approach. The healthcare system alone cannot adequately address all these challenges. A coordinated, cross-sectoral approach, including civil society, social care, and peer support networks is essential to ensuring comprehensive and appropriate care. Currently, guidance on how to support this group remains limited, as their circumstances often fall outside the conventional domains of healthcare, social services, and legal systems. There is a clear need to develop interventions that offer appropriate support to individuals with brain tumors, their families, and the professionals who provide care for this group.

### Future Directions

This qualitative study has contributed to an in-depth understanding of violence related to intracranial tumors, and the findings are intended to provide an exploratory foundation for future research. However, additional studies are needed to further expand knowledge in this area. Plans for quantitative research are already underway, including a questionnaire study and registry-based studies, in order to continue assembling the pieces of this complex puzzle and enable more generalizable conclusions. Future research will hopefully help to identify the factors contributing to violent behavior of patients with an intracranial tumor. There is also a need for research exploring the experiences of professionals, such as healthcare providers and representatives from authorities, who interact with individuals with brain tumors who have perpetrated violence, as well as with family members who have been subjected to such violence. Such studies are currently ongoing.

## Supplementary Material

vdag077_Supplementary_Data

## Data Availability

Data are available upon reasonable request from the authors.
